# Compartment Model and Neural Network-Based Analysis of Combination Medication Ratios

**DOI:** 10.3390/pharmaceutics17020228

**Published:** 2025-02-10

**Authors:** Yuxin Zeng, Jieyu Yang, Yong Li

**Affiliations:** Faculty of Information Engineering and Automation, Kunming University of Science and Technology, Kunming 650500, China; z1079078898@outlook.com (Y.Z.); 18287825744@139.com (J.Y.)

**Keywords:** combined drug therapies, compartment model, neural network, *Cynanchumotophyllum*, *Erigeron breviscapus*

## Abstract

**Background:** Combination medication strategies often involve complex interactions, making determining the appropriate pharmacodynamic component ratios challenging. **Methods:** This study established a time–dose relationship model through the compartment model, deriving the in vivo drug quantity ratios corresponding to the blood concentrations of the pharmacodynamic components. A neural network was then employed to establish a dose–effect relationship model between the blood concentrations of the pharmacodynamic components and the overall body response. Utilizing the feedback adjustment mechanism of neural networks continuously adjusts the network to achieve the desired drug efficacy, thereby deriving the corresponding dose ratio of the pharmacodynamic components. Empirical studies were conducted on combining *Cynanchum otophyllum* saponins *M*_1_ and *M*_2_ with phenobarbital for epilepsy treatment, as well as the anti-ischemic stroke activity of the prototype and metabolites of *Erigeron breviscapus*. **Results:** After adjusting the efficacy, the model recalculated the new ratio proportions for each combination, validated by the reduced Combination Index (*CI*). **Conclusions:** This model provides a new approach to combination medication strategies.

## 1. Introduction

Combined drug therapies often involve the strategic use of multiple agents in specific proportions to target particular diseases [1]. The synergistic or antagonistic interactions between these drugs can enhance the efficacy or reduce side effects, forming the core strategy of combination therapies [2]. However, the complex mechanisms underlying these multi-drug interactions are often difficult to fully elucidate through traditional experimental methods, leading to a gap between theoretical predictions and clinical outcomes [3]. This challenge has sparked growing interest in applying mathematical models to study drug combinations [4]. These models enable the quantitative analysis of drug interactions, improving the precision, predictive power, and control of research outcomes [5]. Several experimental design methods exist for determining the optimal ratios of active components, including orthogonal design, uniform design, baseline equal increment design, and weighted ratio methods. For example, Straetemans et al. [6] applied orthogonal design and significantly improved the systematic evaluation of drug combination experiments. Similarly, Tan et al. [7] explored the use of uniform design measures for determining optimal drug combinations, thereby enhancing the efficiency of drug interaction studies. Additionally, Meadows et al. [8] introduced fixed ratio rays for mixtures, contributing to a better understanding of complex chemical interactions in combination therapies. However, these approaches often struggle to capture complex interaction effects, have optimization limitations, and require extensive experimental conditions and repetitions. As machine learning technologies advance, they are increasingly being applied across various fields, including drug combination therapy. Integrating neural networks with mathematical modeling offers new approaches for predicting therapeutic effects, optimizing dosages, and refining drug formulations.

This paper proposes a dose–effect relationship prediction model combining the compartment model and neural networks. The model predicts drug efficacy by capturing the relationship between component ratios and efficacy within a specific range. Unlike traditional methods, neural networks offer higher computational efficiency by extracting features from one-dimensional data, reducing parameters and improving prediction accuracy. The model optimizes pharmacodynamic component ratios in drug combinations, reducing experimental repetitions and improving efficiency and accuracy through machine learning. Research has shown that *Cynanchum otophyllum* plays a significant role in epilepsy treatment. For instance, Pei et al. [9] demonstrated that in rat models, *Cynanchum otophyllum* significantly counteracted audiogenic seizures. In mice, the plant showed no noticeable effect when used alone in electroshock-induced convulsion tests but exhibited synergistic enhancement when combined with phenobarbital or phenytoin sodium. Additionally, *Cynanchum otophyllum* had no preventive effect on clonic convulsions induced by pentylenetetrazol or convulsions and death caused by strychnine. However, it inhibited avoidance-conditioned reflexes in mice and treated iron sulfate-induced epilepsy in rabbits, aiding in the restoration of abnormal cortical EEGs to normal states. Mu et al. [9] found that in the kindling model of experimental epilepsy in rats, the antiepileptic effects of the total extract of *Cynanchum otophyllum* were close to those of phenobarbital [10]. Clinically, there have been reports of successful epilepsy treatment using *Cynanchum otophyllum* [11].

Phenobarbital (*PHB*) was chosen in this study as it is one of the most widely used antiepileptic drugs in clinical practice, known for its potent sedative and antiepileptic effects. However, *PHB* is also associated with a range of significant side effects, including drowsiness, cognitive impairment, potential for addiction, and hepatotoxicity with prolonged use. These limitations have restricted its broader clinical application and reduced patient compliance. Therefore, exploring combination therapies that can enhance *PHB*’s efficacy while minimizing its dosage and associated side effects is crucial. Research has shown that the main active components of *Cynanchum otophyllum*, saponins *M*_1_ and *M*_2_, when combined with *PHB*, significantly improve its antiepileptic effects, shorten its onset time, and prolong its duration of action, thereby reducing the required *PHB* dosage and its side effects.

Against this background, an empirical study calculated the optimal ratio of saponins *M*_1_ and *M*_2_ to assist *PHB* in inhibiting epilepsy. The results demonstrated that increasing the proportion of *M*_1_ in the *M*_1_ to *M*_2_ ratio further enhanced the antiepileptic efficacy of *PHB*. Additionally, the study explored the anti-ischemic stroke activity of scutellarin compounds and their metabolites from *Erigeron breviscapus*. The combined effect of these three components was greater than that of the original compounds, improving therapeutic outcomes. These findings validate the proposed method for optimizing drug combination strategies.

## 2. Analysis Model of Pharmacodynamic Component Ratios Based on Compartment Model and Neural Networks

### 2.1. Model Structure and Optimization Based on Neural Networks and the Compartment Model

The prediction of the dose–effect relationship is a complex task that requires integrating different models to capture both the drug’s pharmacokinetics and its pharmacodynamics. In this study, the time–dose relationship of the drug in the body is first determined using the compartment model, which divides the biological system into multiple compartments. This model provides a detailed understanding of drug absorption, distribution, metabolism, and elimination. Subsequently, the dose–effect relationship, which describes how the concentration of pharmacodynamic components correlates with drug efficacy, is predicted using neural networks. By combining the time–concentration data from the compartment model with the dose–effect data from the neural network, the optimal drug combination ratio for achieving the desired therapeutic effect can be determined.

Neural networks are used to predict the relationship between the input data, such as the blood concentrations of pharmacodynamic components (e.g., *M*_1_, *M*_2_, and scutellarin), and the output, which is drug efficacy. The neural network is particularly well suited for processing one-dimensional concentration data over time, enabling the model to extract relevant features and make accurate predictions of therapeutic effects. By using neural networks, the model is capable of efficiently handling large datasets, reducing the complexity of traditional methods, and enhancing the prediction accuracy.

The model is trained using historical data to capture the underlying relationships between component concentrations and efficacy. Unlike traditional methods, which rely on predefined factor levels and static processes, neural networks adjust dynamically as new data are introduced. The use of neural networks reduces the number of parameters and enhances the model’s ability to generalize, ensuring reliable predictions across various scenarios.

**Figure 1:**
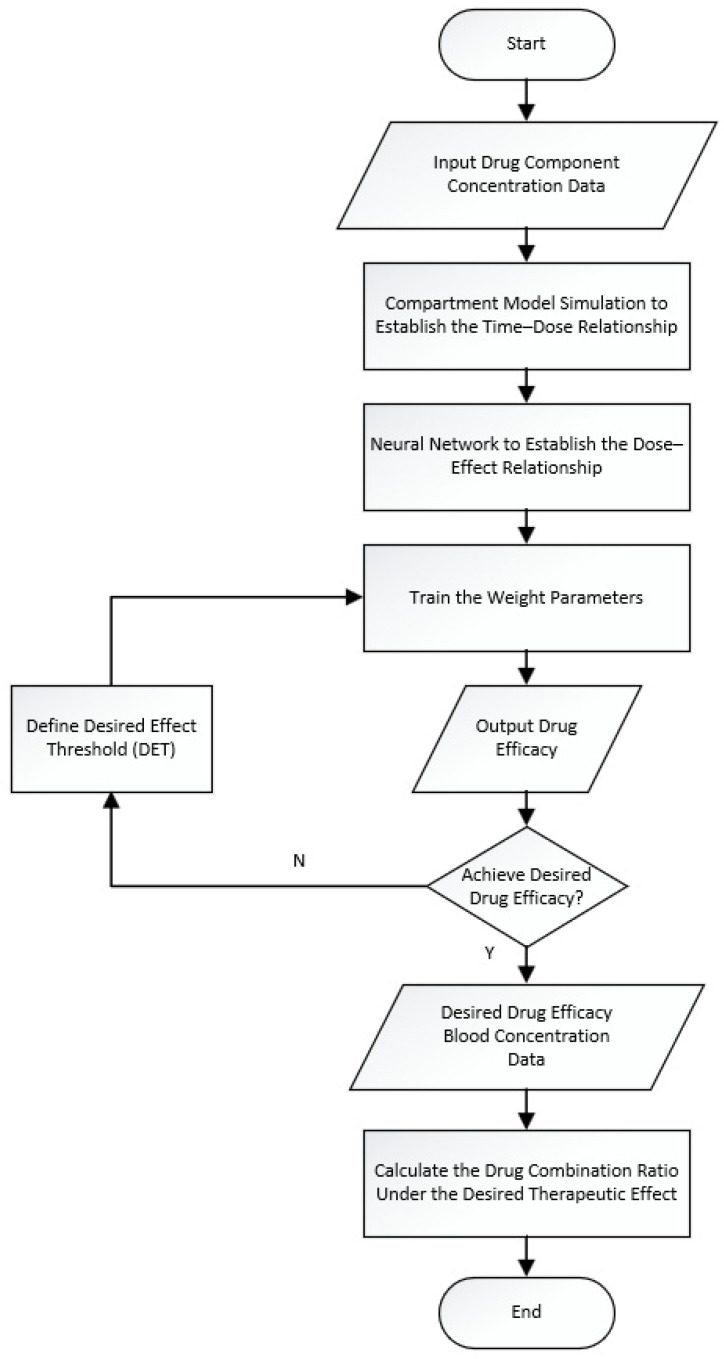
Flowchart for drug efficacy component ratio analysis based on compartment model and HO-1DCNN.

Optimization of the model is performed through the Hippopotamus Optimization Algorithm (HO), which fine-tunes the network’s parameters, such as the learning rate, number of layers, and layer sizes. This optimization process maximizes the model’s performance, enabling it to more accurately capture the complex relationships between component ratios and drug efficacy, thereby improving the reliability of drug combination predictions. After obtaining the stable neural network weight parameters, they are combined with the compartment model to derive the drug component ratio for the desired therapeutic effect. The flowchart in Figure 1 provides an abstract method, with 1D CNN and HO applied for processing one-dimensional concentration data. The flowchart is shown in Figure 1. 

An empirical study was conducted to validate the model’s predictive ability. The study examined the ratio of saponins *M*_1_ and *M*_2_, which assist in inhibiting epilepsy with phenobarbital (*PHB*) while keeping *PHB* constant. The results showed that increasing the proportion of *M*_1_ in the *M*_1_ to *M*_2_ ratio improved the efficacy of *PHB* in inhibiting epilepsy. Additionally, the study explored the anti-ischemic stroke activity of scutellarin compounds and their metabolites derived from *Erigeron breviscapus*. The combined effect of these components exceeded that of the original compounds, demonstrating the model’s effectiveness in optimizing drug combination strategies and improving therapeutic outcomes.

### 2.2. Establishing the Time–Dose Relationship of Pharmacodynamic Components Based on a Single-Compartment Model and Determining the Proportion of In Vivo Drug Quantity

**Figure 2:**
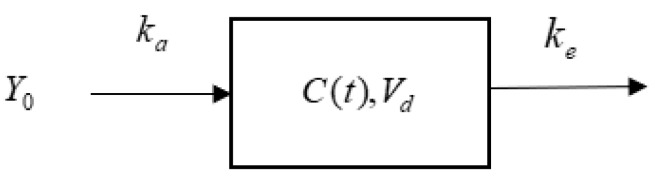
Structure of the single-compartment model.

The compartment model is a commonly used mathematical model in pharmacokinetic analysis. It simulates and describes the processes of drug absorption, distribution, metabolism, and excretion in a biological system. This model is typically represented by first-order linear differential equations characterized by a drug elimination rate proportional to the drug concentration, exhibiting linear kinetic properties. The single-compartment model is the simplest and most commonly used compartment model [12]. Taking the single-compartment model for extravascular administration as an example, its structure is shown in Figure 2.

The time–dose relationship of the single-compartment pharmacokinetic model is as follows:

(1) $$C(t)=\frac{k_{a}F_{BA}Y_{0}}{V_{d}(k_{a}-k_{e})}\left(e^{-k_{a}t}-e^{-k_{e}t}\right)$$

In the formula, C(t) is the plasma concentration at time t, $k_{a}$ is the absorption rate constant, $k_{e}$ is the elimination rate constant, $V_{d}$ is the volume of distribution, $F_{BA}$ is the bioavailability, and is the administered dose.

If there are two or more drug components, the time–concentration relationship of the single-compartment pharmacokinetic model becomes the following:

(2) $$C_{i}(t)=\frac{k_{a_{i}}F_{BA_{i}}Y_{0,i}}{V_{d}\left(k_{a_{i}}-k_{e_{i}}\right)}\left(e^{-k_{a_{i}}t}-e^{-k_{e_{i}}t}\right)$$

In the formula, $C_{i}(t)$ is the plasma concentration of the i pharmacodynamic component at time t, $k_{a_{i}}$ is the absorption rate constant of the i component, $k_{e_{i}}$ is the elimination rate constant, $F_{BA_{i}}$ is the bioavailability of the i component, and $Y_{i}$ is the administered dose of the i-th component.

According to the time–dose relationship of the single-compartment pharmacokinetic model, let $K_{c}=\frac{k_{a_{i}}F_{BA_{i}}Y_{0,i}}{V_{d}\left(k_{a_{i}}-k_{e_{i}}\right)}$, then

(3) $$C_{i}(t)=K_{c_{i}}\left(e^{-k_{a_{i}}t}-e^{-k_{e_{i}}t}\right)$$

Let the in vivo drug quantity corresponding to C(t) be Q(t), then

(4) $$Y_{0,i}=\frac{V_{d}\left(k_{a_{i}}-k_{e_{i}}\right)}{k_{a_{i}}F_{BA_{i}}}K_{c_{i}}$$

If there is another drug component j, then correspondingly have

(5) $$Y_{0{,}_{j}}=\frac{V_{d}\left(k_{a_{j}}-k_{e_{j}}\right)}{k_{a_{j}}F_{BA_{j}}}K_{c_{j}}$$

From Equations (4) and (5), the following results can be derived:

(6) $$\frac{Q_{i}(t)}{Q_{j}(t)}=\frac{K_{c_{i}}(k_{a_{i}}-k_{e_{i}})k_{a_{j}}}{K_{c_{j}}(k_{a_{j}}-k_{e_{j}})k_{a_{i}}}$$

In this equation, $Q_{i}(t)$:$Q_{j}(t)$ represents the ratio of the in vivo drug quantities corresponding to the blood concentrations of drug components i and j. The coefficients on the right side of the equation can be determined by fitting nonlinear least squares. The derivation method described above can also be extended to calculate the in vivo drug quantity ratios for multiple pharmacodynamic components.

### 2.3. Establishing the Dose–Effect Relationship of Pharmacodynamic Components Based on the Neural Network Model and the Desired Efficacy Threshold

A neural network is a machine learning model inspired by the structure of the human brain, designed to recognize patterns in data. It consists of interconnected layers of neurons: an input layer, hidden layers, and an output layer. Each neuron processes input data through weighted connections and applies an activation function to produce output. During training, the network adjusts these weights using backpropagation to minimize errors between the predicted and actual values. Neural networks are versatile and can handle tasks like classification, regression, and pattern recognition.

Combining the compartment model and neural networks allows for the optimization of the drug dosage and ratio adjustments. The compartment model simulates the processes of drug absorption, distribution, and elimination in the body, providing time-varying drug concentration data. These data are calculated through time–dose relationship equations, yielding insights into the drug’s metabolic process in vivo. Neural networks learn from the time–dose data generated by the compartment model, capturing pharmacodynamic characteristics at various time points. By integrating the compartment model with neural networks, it becomes possible to optimize the therapeutic effects of drug combinations by adjusting the pharmacodynamic component ratios in real time based on these data.

Based on the initial dose–effect relationship model of pharmacodynamic components, the corresponding drug effects are predicted by inputting the blood concentrations of the pharmacodynamic components. During the efficacy optimization process of the neural network, a “Desired Effect Threshold” (DET) is introduced into the program to ensure that the output matches the expected efficacy state more closely. This threshold dynamically adjusts the model’s prediction results to optimize drug efficacy. Unlike traditional methods, such as orthogonal or uniform design, which require all factor levels to be predefined before the experiment and cannot be adjusted during the process, the neural network can dynamically change the prediction threshold based on model performance during training. Traditional methods are often suitable for handling linear or simple multi-factor interactions. In contrast, the neural network can learn and express nonlinear patterns between drug effects, leading to more precise optimization.

At the beginning of the model, the DET is initialized to a predefined value, usually determined based on the training set. During each prediction process, the prediction values output by the neural network model are compared with the DET. If the model’s prediction value exceeds the DET, the current drug concentration has achieved the desired efficacy, and the prediction is classified as 1 (effective). If the prediction value is below the DET, it indicates that the efficacy has not met expectations, and the prediction is classified as 0 (ineffective). After each iteration, the neural network performs backpropagation based on the classification results of the DET, adjusting the model parameters by calculating the loss function (e.g., binary cross-entropy loss) to optimize the prediction results.

The DET can be dynamically adjusted based on model performance during training. For example, if the model’s prediction accuracy improves, the DET can be gradually increased to optimize the efficacy further. Conversely, if the model’s performance is poor, the DET can be lowered to increase the model’s sensitivity. The optimization algorithm initializes the weights during global and local searches and searches for the optimal learning rate and iteration count based on different DET settings, adjusting the contribution weights of the input parameters in the neural network model. After multiple iterations, the neural network alters the predicted drug efficacy results, with these concentrations corresponding one-to-one with the desired efficacy improvement. After the efficacy changes, the in vivo drug quantity corresponding to the blood concentration also changes. Based on the contribution weights from the neural network’s backpropagation after the efficacy change, the weights are normalized to obtain the in vivo drug quantity corresponding to the blood concentration after the efficacy change.

At this point, the proportion of the in vivo drug quantities for the components corresponding to the efficacy change becomes the following:

(7) $$\frac{{Q^{\prime }}_{i}(t)}{{Q^{\prime }}_{j}(t)}=\frac{{K^{\prime }}_{c_{i}}({K^{\prime }}_{a_{i}}-{K^{\prime }}_{e_{i}}){K^{\prime }}_{a_{j}}}{{K^{\prime }}_{c_{j}}({K^{\prime }}_{a_{j}}-{K^{\prime }}_{e_{j}}){K^{\prime }}_{a_{i}}}$$

After fitting the coefficients on the right side of the equation, the in vivo drug quantity ratio corresponding to the blood concentration after the efficacy change can be obtained. Finally, by relating the in vivo drug quantity ratio to the initial efficacy proportions, the new proportions of each component after the efficacy change can be calculated.

## 3. Experimental Section

Phenobarbital (*PHB*) is a commonly used clinical drug for treating convulsive epilepsy [13]. *Cynanchum otophyllum* has shown good efficacy in treating epilepsy, chronic hepatitis, urticaria, and other conditions [14]. The main components of *Cynanchum otophyllum* are saponins *M*_1_ (Qingyangshengenin 3-O-β-D-oleandropyranosyl-(1→4)-β-D-cymaropyranosyl-(1→4)-β-D-digitoxopyranoside) and *M*_2_ (Qingyangshengenin 3-O-β-D-oleandropyranosyl-(1→4)-β-D-cymaropyranosyl-(1→4)-β-D-digitoxopyranosyl-(1→4)-β-D-cymaropyranoside), which exhibit a synergistic inhibitory effect on epilepsy when used in combination with *PHB* [15]. The components *M*_1_ and *M*_2_ in *Cynanchum otophyllum* can enhance the antiepileptic efficacy of *PHB*, extend its duration of action, and shorten its onset time. Clinically, *Cynanchum otophyllum* tablets can assist *PHB* in treating epilepsy, but the specific ratio of its active components *M*_1_ and *M*_2_ on drug efficacy requires further investigation. This study verifies the feasibility of the proposed calculation method by analyzing the efficacy component ratios of saponins *M*_1_ and *M*_2_ in assisting phenobarbital in inhibiting epilepsy.

### 3.1. Experiment and Preparation of Test Solutions

Mice were purchased from Hunan Songji Jingda Laboratory Animal Co., Ltd., Changsha, China. (License No.: SCXK(Yunnan)K2013-003), with a body weight of 15–22 g. They were housed at 25 °C and 60% relative humidity, provided with regular feed and water, and allowed to acclimate for 3 days before the experiment. *Cynanchum otophyllum* was purchased from the Kunming Chinese Herbal Medicine Market. *PHB* was purchased from Shanghai Xinya Pharmaceutical Co., Ltd., Shanghai, China. The animal experiments were reviewed and approved by the Animal Ethics Committee of Kunming University of Science and Technology.

Saponins *M*_1_ and *M*_2_ were extracted from *Cynanchum otophyllum* [15] and prepared with 2% Tween 80 solution, while *PHB* was prepared with physiological saline. When *PHB* was used alone, the dosage was 2 mg/kg. When *PHB* was combined with *M*_1_ and *M*_2_, the dosages were 2 mg/kg, 12 mg/kg, and 6 mg/kg [15], respectively.

### 3.2. Experimental Data Collections

Physiological saline was applied to the ears of 128 mice, and their ears were clamped with alligator clip electrodes for continuous electroshock, conducting the maximal electroshock (MES) induction experiment. The mice were then divided into 8 large groups, each containing 16 mice, and further subdivided into 2 smaller groups with 8 mice each (8 mice per time point). The mice were administered *PHB* alone or in combination with *M*_1_ and *M*_2_ via gavage. The convulsive responses of the mice were observed over a period of 0.17 to 24 h: if the hind limbs did not exhibit tonic convulsions after drug administration, it indicated that the drug had an antiepileptic effect; if tonic convulsions occurred, the drug was considered ineffective. If any mice died, their data were excluded from the experiment. Plasma drug concentrations of the components were measured using LC-MS/MS multi-component quantification technology [15], and the results were expressed as mean ± standard deviation. The plasma concentrations and convulsive responses for *PHB* administered alone and in combination with *M*_1_ and *M*_2_ are shown in Table 1 and Table 2. The inhibitory rate, which was defined as the probability of an anticonvulsive response at a specific time point, was used as the pharmacodynamic evaluation indicator for *Cynanchum otophyllum* in inhibiting epilepsy. The inhibitory rate was calculated as the number of mice without convulsive response after treatment divided by the total number of mice with and without convulsive response.

### 3.3. Data Preprocessing

Due to the high cost of obtaining experimental data and the limited sample size, dividing the data into training and test sets is challenging, resulting in poor prediction robustness for machine learning methods with small samples. To address this issue, this study utilized the Time Generative Adversarial Network (TimeGAN) [16] to effectively augment the original data, enhancing the learning capacity of the neural network. TimeGAN is a deep learning model specifically designed to generate realistic time-series data. It combines the framework of Generative Adversarial Networks (GANs) with a recurrent network architecture, enabling it to capture both temporal dependencies and feature correlations within the data. The model consists of four main components: an embedding network for learning data representations, a generator to create synthetic time-series data, a discriminator to distinguish real from synthetic data, and a supervised loss to ensure the generated data match the temporal structure of the original data. During training, the generator learns to produce synthetic data samples that closely mimic the patterns and statistical properties of the real data, while the discriminator learns to differentiate between real and synthetic samples.

In this study, TimeGAN was trained on the time-series data from Table 2 to learn the underlying patterns and relationships among the pharmacodynamic components (e.g., *M*_1_, *M*_2_, and *PHB* plasma concentrations). Once trained, TimeGAN generated synthetic samples by extrapolating plausible variations in the original data while preserving key statistical properties, such as mean, variance, and temporal dependencies. Using the TimeGAN method, the data from Table 2 were augmented, resulting in a total of 792 datasets, as shown in Table 3. The augmented data maintained the same structure as the original data and were validated for consistency using Pearson correlation analysis and statistical tests. The results confirmed that the synthetic data closely aligned with the characteristics of the original dataset.

Using Python tools, Pearson correlation analysis and two-tailed significance tests were conducted on the data before and after augmentation. In the original data, the correlation coefficients between the *PHB* concentration and *M*_1_ concentration, *M*_2_ concentration, time, and MES response were −0.570, −0.572, 0.643, and −0.327, respectively, with *p*-values of 5.18 × 10^−184^, 1.95 × 10^−185^, 1.78 × 10^−248^, and 2.33 × 10^−54^. Overall, the *PHB* concentration showed significant correlations with all four variables (*p* < 0.05).

In the augmented data, the correlation coefficients between the *PHB* concentration and *M*_1_ concentration, *M*_2_ concentration, time, and MES response were 0.576, 0.418, −0.473, and 0.274, respectively, with *p*-values of 4.96 × 10^−77^, 1.13 × 10^−37^, 2.91 × 10^−49^, and 2.68 × 10^−16^. The augmented data also demonstrated statistically significant correlations between the *PHB* concentration and all four variables (*p* < 0.05).

Overall, the correlation coefficients between variables in the original and augmented data showed minimal changes. The linear relationships between the *PHB* concentration and *M*_1_, *M*_2_, time, and MES remained consistent, with no significant differences observed. This indicates that the data augmentation effectively preserved the original correlation characteristics, supporting further analysis.

### 3.4. Establishing the Dose–Effect Relationship of Pharmacodynamic Components Based on the HO-1 DCNN Model

**Figure 3:**
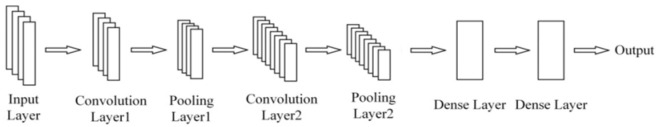
One-dimensional convolutional neural network structure.

A one-dimensional convolutional neural network (1D CNN) is a deep learning model that processes one-dimensional data. It captures local features by sliding a convolutional kernel over the data and uses pooling layers to reduce the feature dimensions while retaining important information. During training, the model optimizes its parameters through a backpropagation algorithm to minimize prediction errors. The typical structure of a 1D CNN is shown in Figure 3, where convolutional and pooling layers are alternately stacked to gradually extract features from the input data, followed by fully connected layers for further processing and integration of features, ultimately providing prediction results in the output layer.

The Hippopotamus Optimization Algorithm (HO) is inspired by the physiological behavior of hippos [17], simulating their movement in water for global search and on land for local search. The male hippo’s position is updated as follows:

(8) $$X_{i}^{M_{hippo}}:X_{ij}^{M_{hippo}}=x_{ij}+y_{1}\cdot (D_{hippo}-I_{1}x_{ij})$$

In the exploration phase, hippos update their positions in the river or pond based on their competitive habits within the population.

(9) $$X_{i}^{FB_{hippo}}:x_{ij}^{FB_{hippo}}=\left\{\begin{array}{l} x_{ij}+h_{1}\cdot (D_{hippo}-I_{2}MG_{j}),\\ E \end{array}\right.\begin{array}{c} T>0.6\\ else \end{array}$$

(10) $$E=\left\{\begin{array}{l} x_{ij}+h_{2}\cdot (MG_{j}-D_{hippo})r_{6},\\ l_{j}+r_{7}\cdot (u_{j}-l_{j}), \end{array}\right.\begin{array}{c} T>0.6\\ else \end{array}$$

(11) $$X_{i}=\left\{\begin{array}{l} X_{i}^{FB_{hipoo}},\\ X_{i}, \end{array}\right.\begin{array}{c} F_{i}^{FB_{hippo}}<F_{i}\\ else \end{array}$$

(12) $$X_{i}=\left\{\begin{array}{l} X_{i}^{M_{hipoo}},\\ X_{i}, \end{array}\right.\begin{array}{c} F_{i}^{M_{hippo}}<F_{i}\\ else \end{array}$$

$X_{i}^{M_{hippo}}$ represents the position of the male hippo and $D_{hippo}$ represents the position of the dominant hippo (the hippo with the best fitness in the current network model iteration). Equations (9) and (10) describe the position of female or immature hippos within the population ($FB_{hippo}$). In Equations (11) and (12), the positions of juvenile hippos are updated after moving away from the population, and their fitness $F_{i}$ is compared. During the exploration phase, based on the threshold T, a global or local search is determined to enhance the adaptability of the algorithm. In the defense phase, hippos defend themselves by emitting threatening sounds towards predators.

(13) $$\mathrm{Pr}edator_{j}=l_{j}+r_{8}\cdot (u_{j}-l_{j}),j=1,2,..,m$$

(14) $$\vec{D}=|\mathrm{Pr}edator_{j}-x_{ij}|$$

(15) $$\left\{\begin{array}{l} \vec{R}L⊕\mathrm{Pr}edator_{j}+(\frac{1}{c-d\times \cos(2\pi g)})\cdot (\frac{1}{\vec{D}}),\\ \vec{R}L⊕\mathrm{Pr}edator_{j}+(\frac{1}{c-d\times \cos(2\pi g)})\cdot (\frac{1}{2\times \vec{D}+r_{9}}), \end{array}\right.\begin{array}{c} F_{predator}<F_{i}\\ F_{predator}\geq F_{i} \end{array}$$

Equation (13) represents the position of the predator in the search space. Equation (15) represents the distance between the $F_{predator}$ hippo and the predator. Hippos adopt different defense behaviors based on their distance from the predator. The first and second stages complement each other, effectively reducing the risk of getting trapped in local optima and enhancing the HO algorithm’s exploitation ability in local searches, thereby improving optimization results.

The Hippopotamus Optimization Algorithm (HO) was chosen for its superior ability to perform global and local searches effectively. During global search, hippos update their positions in a probabilistic manner based on water movement dynamics, while local search simulates land movement to refine solutions. The HO algorithm also incorporates a predator-avoidance mechanism, which enhances its ability to escape local optima. Compared to Adam and RMSprop, HO demonstrated faster convergence and higher stability in handling complex nonlinear optimization problems, as detailed in Section 4.2.

The Hippopotamus Optimization Algorithm (HO) combines a one-dimensional convolutional neural network (1DCNN) to analyze pharmacodynamic components and optimize their ratios. Blood concentrations are input into the 1DCNN, which outputs the drug effects. The HO algorithm optimizes the model parameters, establishing a dose–effect relationship. Metrics such as accuracy, precision, recall, and F1-score ensure the model accurately reflects the relationship between pharmacodynamic components and their effects, providing a high-precision and robust solution.

## 4. Results and Discussion

### 4.1. Analysis of Initial Proportion Relationships of Pharmacodynamic Components In Vivo

Interactions may occur when multiple drugs are used simultaneously, affecting the overall efficacy. These interactions are primarily categorized into two types: synergistic and antagonistic effects. Synergistic effects refer to the enhanced efficacy observed when two or more drugs are used together, where one drug boosts the therapeutic effect of another. Conversely, antagonistic effects occur when the combined use of drugs weakens each other’s effects or one drug diminishes the efficacy of another. In pharmacokinetic studies, the Combination Index (*CI*) is used to evaluate the interaction between drugs, whether synergistic or antagonistic. The *CI* is calculated based on the Chou–Talalay method using the following formula:

(16) $$CI=\frac{{\left(D_{1}\right)}_{combo}}{{\left(D_{1}\right)}_{alone}}+\frac{{\left(D_{2}\right)}_{combo}}{{\left(D_{2}\right)}_{combo}}$$

In this context, ${\left(D_{1}\right)}_{combo}$ and ${\left(D_{2}\right)}_{combo}$ represent the doses used in combination therapy, while ${\left(D_{1}\right)}_{alone}$ and ${\left(D_{2}\right)}_{combo}$ are the doses required to achieve the same effect when each drug is used individually. The *CI* can determine whether the drug combination exhibits synergistic effects (*CI* < 1), additive effects (*CI* = 1), or antagonistic effects (*CI* > 1). 

The applicability of the Combination Index (*CI*) in vivo deserves further analysis, as its calculation is traditionally based on in vitro experiments, which are less complex than in vivo systems. In vivo studies involve additional factors such as metabolism, drug distribution, immune responses, and tissue-specific drug dynamics, which may significantly alter drug interactions and make the interpretation of the *CI* more challenging.

Zimmer et al. (2017) [18] highlighted that heterogeneity in drug distribution and metabolism is a key factor influencing the *CI* in in vivo studies. By integrating pharmacokinetic data and time-dependent modeling, their study demonstrated that the transient nature of antagonistic effects (*CI* > 1) could be better understood and resolved. For instance, instances of *CI* > 1 may arise due to competitive inhibition of drug targets or metabolic pathway saturation, where one drug reaches its peak concentration while the other is still being metabolized, leading to temporary antagonism. These findings suggest that time-dependent pharmacokinetic modeling could improve the accuracy and reliability of the *CI* in complex biological systems.

The results of this study also align with these observations. The transient *CI* > 1 values observed at specific time points could be explained by the competitive dynamics between the drug components and their differing pharmacokinetics. Such variations emphasize the need for additional temporal and pharmacokinetic analyses when applying the *CI* in vivo. By integrating these approaches, the *CI* can remain a valuable tool for evaluating drug synergy under in vivo conditions despite the increased complexity of biological systems. By calculating the *CI* using the data from Table 3, the impact of combining *PHB* with *M*_1_ and *M*_2_ on the efficacy of *PHB* can be assessed. The calculated *CI* values are shown in Table 4.

The results show that when *M*_1_ and *M*_2_ are used in combination with *PHB*, the *CI* is less than 1 for most time points except during the early stages of drug action at 0.17 h and 0.5 h, indicating a significant synergistic effect between *M*_1_, *M*_2_, and *PHB*. This suggests that the combined use of *PHB* with *M*_1_ and *M*_2_ significantly enhances the efficacy of *PHB* compared to using *PHB* alone, demonstrating a synergistic effect between *M*_1_, *M*_2_, and *PHB*. Based on the relationship between the blood concentration and time in the one-compartment pharmacokinetic model, the data from Table 3 were used, and the pharmacokinetic models for *M*_1_ and *M*_2_ under the initial ratio were obtained by fitting with nonlinear least squares. The time–dose relationships are expressed in Equation (12) and Equation (13), respectively, with regression analysis yielding determination coefficients (R^2^) of 0.876 and 0.838, indicating a good fit for the model.

(17) $$C_{M1}(t)=5.186(e^{-0.072t}-e^{-11.457t})$$

(18) $$C_{M2}(t)=0.409(e^{-0.198t}-e^{-11.398t})$$

The fitted values for *M*_1_’s $k_{a}$, $k_{e}$, and $C_{\infty }$ are 11.457, 0.072, and 5.186, respectively. For *M*_2_, the $k_{a}$, $k_{e}$, and $C_{\infty }$ values are 11.398, 0.198, and 0.409, respectively. Using Equation (6), the in vivo drug quantity ratio corresponding to the blood concentration when the efficacy remains unchanged is calculated to be 12.83:1.

### 4.2. HO-1DCNN Model Evaluation

Using the data from Table 3, *M*_1_*, M*_2_, and *PHB* blood concentrations were used as the input, and the efficacy as the output, to predict the corresponding efficacy under various blood concentrations using 1DCNN, establishing a dose–effect relationship model under the initial efficacy conditions. The HO algorithm was employed with a population size of 30, a learning rate range of [0.00001, 0.01], and an iteration range of [100, 1000]. Among the data, 671 sets were used as the training set, and 119 sets as the test set, resulting in the optimal learning rate of 0.00001 and 213 iterations. Through this optimization, the model achieved a precision, recall, F1-score, and accuracy of 0.91, 0.93, 0.92, and 0.96, respectively. The results indicate that the network’s contribution weights accurately reflect the influence of *M*_1_*, M*_2_*,* and *PHB* blood concentrations on efficacy, and the model effectively describes the relationship between the blood concentration and drug efficacy (dose–effect relationship).

### 4.3. Analysis of Pharmacodynamic Component Ratios After Efficacy Improvement

Building on the dose–effect model established in Section 3.2 under the initial efficacy conditions, an optimal efficacy threshold was introduced during prediction to adjust the efficacy, aiming to output more effective pharmacodynamic component states. A threshold was set for ineffective states to convert some ineffective states into effective ones through adjustment, thereby increasing the proportion of effective outputs and improving the overall efficacy. The optimal efficacy threshold dynamically adjusts the model output during optimization to achieve the desired efficacy. The contribution weights of *M*_1_, *M*_2_, and *PHB* blood concentrations to the efficacy were then updated through the backpropagation process in 1DCNN, allowing the calculation of the blood concentrations after efficacy improvement and establishing a dose–effect model under improved efficacy conditions. The blood concentrations of each component, MES responses, and inhibition rates after efficacy improvement are shown in Table 5.

The blood concentrations after efficacy improvement were used as the input for the HO-1DCNN model and the 1DCNN and CatBoost network models to predict the improved efficacy. After training, the loss function errors of all models tended to converge. Table 6 shows the evaluation metrics for different dose–effect models after efficacy improvement. It can be observed that all three models performed well in predicting the improved efficacy, with the efficacy predicted by the 1DCNN and CatBoost models showing a high degree of consistency with the efficacy adjusted by the HO-1DCNN.

To validate the performance of the HO-1DCNN network, this study first compared it with traditional optimization algorithms such as Adam and RMSprop, which are widely used for neural network training. As shown in Table 7, the HO-1DCNN outperformed Adam-1DCNN and RMSprop-1DCNN in both the prediction accuracy and prediction time. Specifically, the HO-1DCNN achieved a 91% accuracy and 0.50 min prediction time, compared to Adam’s 85% accuracy and 0.70 min prediction time. These results highlight the effectiveness and efficiency of the HO algorithm, particularly in handling complex nonlinear optimization problems.

To further demonstrate the advantages of HO-1DCNN, this study also compared it with nature-inspired optimization algorithms, including the Whale Optimization Algorithm (WOA)-optimized 1DCNN neural network (WOA-1DCNN) [19], the Sparrow Search Algorithm (SSA)-optimized 1DCNN neural network (SSA-1DCNN) [20], and the Gray Wolf Optimizer (GWO)-optimized 1DCNN neural network (GWO-1DCNN) [21]. The improved blood concentrations were input into these four models for efficacy prediction, and the prediction accuracy and prediction time were recorded. As shown in Table 7, HO-1DCNN achieved a higher prediction accuracy and efficiency compared to SSA-1DCNN, WOA-1DCNN, and GWO-1DCNN, further emphasizing its robustness and performance advantages.

Using the data from Table 5, the nonlinear least squares method was employed to fit the single-compartment pharmacokinetic models for *M*_1_ and *M*_2_ after the efficacy was increased. The time–dose relationships post-efficacy enhancement are expressed in Equation (14) and Equation (15), respectively. The regression analyses’ coefficients of determination (R^2^) were 0.838 and 0.768, indicating a good fit for the models.

(19) $$C_{M1}(t)=4.431(e^{-0.351t}-e^{-5.172t})$$

(20) $$C_{M2}(t)=0.279(e^{-0.201t}-e^{-5.744t})$$

**Figure 4:**
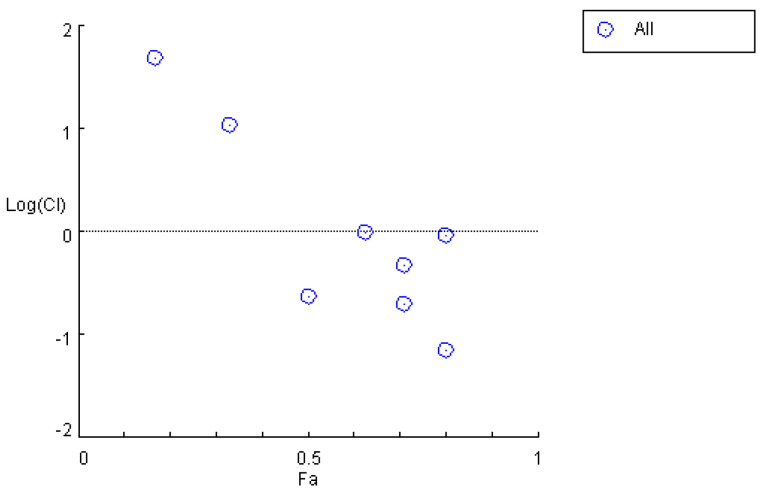
*CI* graph for the combined use of *M*_1_, *M*_2_, and *PHB*.

**Figure 5:**
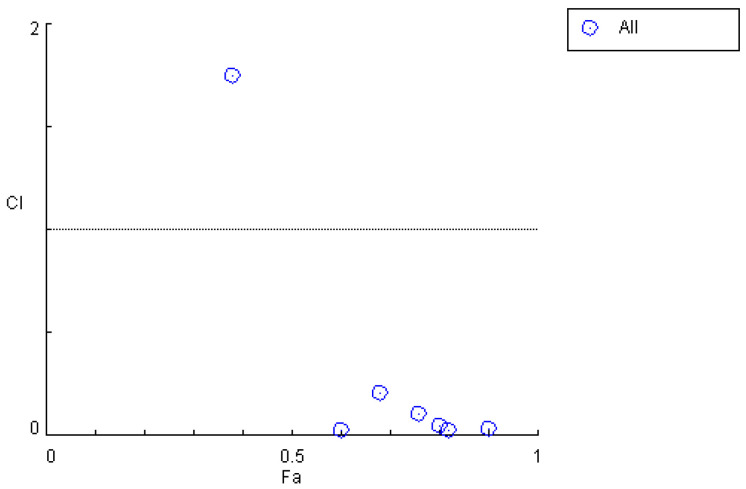
*CI* plot for the combined use of *M*_1_, *M*_2_, and *PHB* after efficacy enhancement.

Through fitting, the $k_{a}$, $k_{e}$, and $C_{\infty }$ values for *M*_1_ were determined to be 5.172, 0.351, and 4.431, respectively. For *M*_2_, the $k_{a}$, $k_{e}$, and $C_{\infty }$ values were 5.744, 0.201, and 0.279, respectively. Using Equation (10), the ratio of the in vivo drug amounts corresponding to the blood concentrations of *M*_1_ and *M*_2_ was calculated to be 15.39:1 when the inhibition rate was increased. At this point, based on the blood concentrations obtained after a 5% efficacy increase, The *CI* values were calculated as shown in Table 8:

When the in vivo drug quantity ratio corresponding to the blood concentration was 12.83:1, the corresponding dosages of *M*_1_ and *M*_2_ were 12 mg/L and 6 mg/L, respectively, indicating an initial ratio of 2:1. Accordingly, after the enhancement in the efficacy of *Cynanchum otophyllum*, when the in vivo drug quantity ratio corresponding to the blood concentrations of *M*_1_ and *M*_2_ was 15.39:1, the ratio of *M*_1_ to *M*_2_ was determined to be 2.39:1. By calculating the *CI*, the data in Table 4 and Figure 4 were obtained, showing the results before efficacy enhancement. Compared to these, Table 8 and Figure 5 demonstrate that the *CI* significantly decreased across all periods after efficacy enhancement. This validates that increasing the dosage of *M*_1_ and *M*_2_ results in a stronger synergistic interaction between *M*_1_,*M*_2_, and *PHB.*

To verify the generalizability of the model, a study was conducted using breviscapine and scutellarein compounds from *Erigeron breviscapus*, focusing on the quantitative relationship of multi-source convergence phenomena in rats and the anti-ischemic stroke activity of the prototype and its metabolites. When breviscapine (*Bre*) and scutellarein (*Scu*) were combined, the metabolic product hispidulin (*His*) was produced. The combination of all three components showed greater therapeutic efficacy for stroke treatment than individual or dual-component treatments.

When breviscapine (*Bre*) was used alone, the doses were 40 mg/kg (0.09 mM/kg) and 20 mg/kg (0.04 mM/kg). For the combined administration, breviscapine (*Bre*) at 40 mg/kg and scutellarin (*Scu*) at 24.8 mg/kg (0.17 mM/kg) were prepared with normal saline.

Healthy mice were selected and randomly divided into three groups. The three groups were administered the following treatments via intraperitoneal injection: breviscapine (*Bre*) 40 mg/kg (0.09 mM/kg) and 20 mg/kg (0.04 mM/kg); breviscapine (*Bre*) 20 mg/kg (0.04 mM/kg); and a combination of breviscapine (*Bre*) 40 mg/kg and scutellarin (*Scu*) 24.8 mg/kg (0.17 mM/kg). The Morris water maze test was used to evaluate the effects of the prototype and metabolites of scutellarin compounds in *Erigeron breviscapus* on mice’s learning and memory abilities. The treatments were administered once daily for 10 consecutive days, with training conducted 2 h after administration on the 9th day. The plasma concentrations of each component and the number of correct responses by mice under the combined administration are shown in Table 9.

Data were expanded using the same experimental method as for *Cynanchum otophyllum* to obtain Table 10.

The corresponding relationship between the inhibition rate and the *CI* value can also be obtained from these data, as shown in Table 11.

The results show that when *Bre* and *Scu* are combined with *His*, the *CI* is less than 1 for most time points except during the early stages of drug action at 0.25 h, indicating a significant synergistic effect between *Bre*, *Scu*, and *His*. This suggests that the combined use of *Bre*, *Scu*, and *His* significantly enhances the efficacy compared to the use of each drug individually, demonstrating a synergistic effect between *Bre*, *Scu*, and *His*. Based on the relationship between the blood concentration and time in the one-compartment pharmacokinetic model, the data from Table 10 were used, and the pharmacokinetic models for *Bre* and *Scu* under the initial ratio were obtained by fitting with nonlinear least squares. The time–dose relationships are expressed in Equation (13) and Equation (14), respectively, with regression analysis yielding determination coefficients (R^2^) of 0.783 and 0.868, indicating a good fit for the model.

(21) $$C_{Bre}(t)=116.443(e^{-0.221t}-e^{-1.647t})$$

(22) $$C_{Scu}(t)=197.8(e^{-0.065t}-e^{-0.903t})$$

The fitted values for *Bre*’s $k_{a}$, $k_{e}$, and $C_{\infty }$ are 1.647, 0.221, and 116.443, respectively. For *Scu*, the $k_{a}$, $k_{e}$, and $C_{\infty }$ values are 0.903, 0.065, and 197.8, respectively. Using Equation (6), the in vivo drug quantity ratio corresponding to the blood concentration when the efficacy remains unchanged is calculated to be 0.543:1.

The model used in this study is consistent with the previous prediction model of *Cynanchum otophyllum*, employing a similar architecture and approach. The primary difference lies in the incorporation of an additional interaction feature module, which enhances the predictive capability by accounting for new variables in the dose–effect relationship. Using the blood concentrations of *Bre*, *Scu*, and *His* as the input and the efficacy as the output, a 1DCNN was employed to predict the corresponding efficacy under different blood concentrations, establishing a dose–effect relationship model under the initial efficacy conditions. Compared to the prediction model of *Cynanchum otophyllm*, this model introduces a new interaction feature module. Based on the quantitative research on the multi-targeted metabolic phenomena of *Erigeron breviscapus* scutellarin compounds in vivo, it is observed that when breviscapine and scutellarin are co-administered, a portion of breviscapine is converted into scutellarin and hispidulin.

By adjusting the desired efficacy threshold, the expected efficacy was achieved. The contribution weights of *Bre*, *Scu*, and *His* blood concentrations were updated through the backpropagation process in the 1DCNN, and a dose–effect model was established under improved efficacy conditions. After the efficacy improvement, the blood concentrations of each component and MES response inhibition rates are shown in Table 12.

Similarly, after the efficacy was enhanced, the nonlinear least squares method was used to fit the single-compartment pharmacokinetic models for *Bre* and *Scu*. The time–dose relationships post-efficacy enhancement are expressed in Equation (17) and Equation (18), respectively. The regression analyses’ coefficients of determination (R^2^) were 0.751 and 0.812, indicating a good fit for the models.

(23) $$C_{Bre}(t)=34.315(e^{-0.111t}-e^{-3.064t})$$

(24) $$C_{Scu}(t)=125.667(e^{-0.106t}-e^{-0.052t})$$

**Figure 6:**
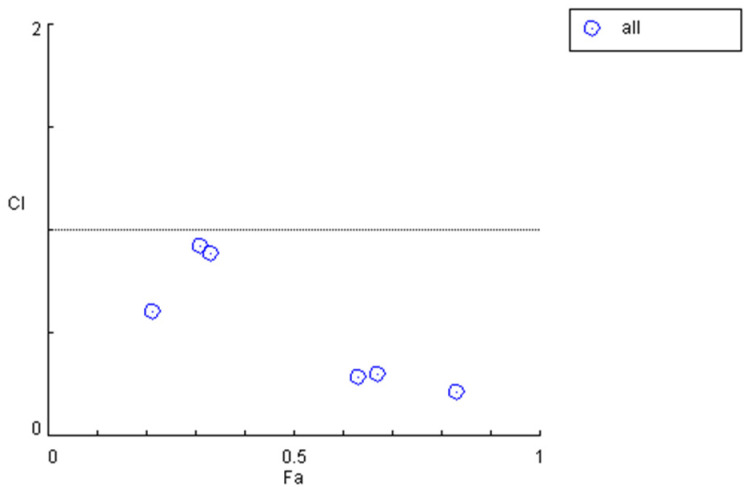
*CI* graph for the combined use of *Bre*, *Scu*, and *His*.

**Figure 7:**
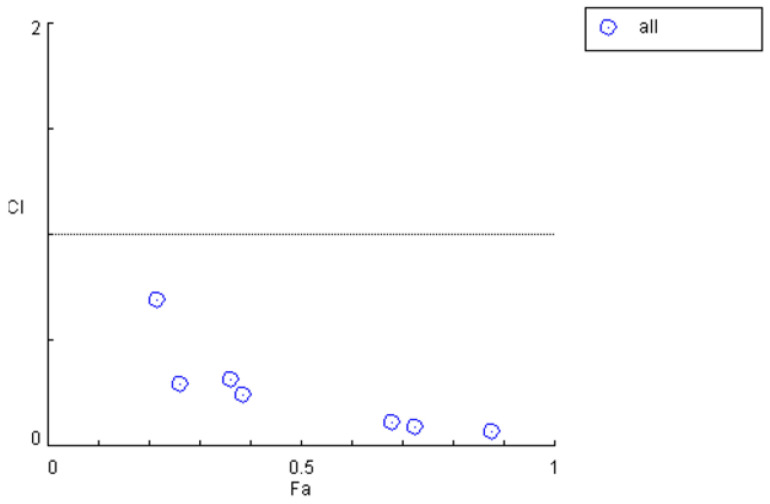
*CI* plot for the combined use of *Bre*, *Scu*, and *His* after efficacy enhancement.

Through fitting, the $k_{a}$, $k_{e}$, and $C_{\infty }$ values for *Bre* were determined to be 3.064, 0.111, and 34.315, respectively. For *Scu*, the $k_{a}$, $k_{e}$, and $C_{\infty }$ values were 0.052, 0.106, and 125.667, respectively. Using Equation (7), the ratio of the in vivo drug amounts corresponding to the blood concentrations of *Bre* and *Scu* was calculated to be 0.334:1 when the inhibition rate was increased. At this point, based on the blood concentrations obtained after a 5% efficacy increase, the *CI* values were calculated as follows:

In the *Erigeron breviscapus* group, when the in vivo drug quantity ratio corresponding to the blood concentration was 0.543:1, the corresponding dosages of *Bre* and *Scu* were 40 mg/kg and 24.8 mg/kg, respectively, indicating an initial ratio of 1.61:1. Accordingly, after the efficacy of the combined administration of the three drugs improved, when the in vivo drug quantity ratio corresponding to the blood concentrations of *Bre* and *Scu* was 0.334:1, the ratio of *Bre* to *Scu* was determined to be 0.992:1. By calculating the *CI*, the data in Table 11 and Figure 6 were obtained, showing the results before efficacy enhancement. Compared to these, Table 13 and Figure 7 demonstrate that the *CI* significantly decreased across all time periods after efficacy enhancement. This validates that increasing the dosage of *Scu* in the *Bre* to *Scu* ratio strengthens the synergistic interaction between *Bre*, *Scu*, and *His*.

By utilizing machine learning tools and the compartment model, this study mapped the original drug combinations and blood concentrations to the efficacy, creating a visual model to show how drug ratios should be adjusted after efficacy enhancement. The models began with an initial ratio of 2:1 for *M*_1_ and *M*_2_ and 1.61:1 for *Bre* and *Scu*. By adjusting the optimal efficacy threshold within the 1DCNN, the contribution weights of the input parameters were modified through backpropagation. After normalizing these weights, it was found that when the efficacy improved by 5%, the ratio of *M*_1_ and *M*_2_ adjusted to 2.39:1, while the ratio of *Bre* to *Scu* adjusted to 0.992:1. A comparison of the *CI* before and after efficacy enhancement showed that, for both *M*_1_ and *M*_2_ as well as *Bre* and *Scu*, the optimized drug ratios resulted in improved efficacy. This method provides a generalized new approach for exploring the nonlinear relationship between efficacy and drug ratios.

## 5. Conclusions

This study established a time–dose relationship for pharmacodynamic components using a compartment model, deriving the in vivo drug quantity ratios corresponding to the blood concentrations. Combining the neural network and the compartment model effectively captures the complex nonlinear relationships between drug combination ratios and their effects. Under limited experimental conditions, this approach can quickly determine the pharmacodynamic component ratios corresponding to efficacy improvements, accounting for the complex interactions between drugs. Using *Cynanchum otophyllum* and *Erigeron breviscapus* as examples, the empirical analysis demonstrates that the results obtained through this method align well with the experimental conclusions. This integration reduces the number of experiments, increases the robustness of the results, and addresses the limitations of traditional methods such as orthogonal design, uniform design, baseline equal increment design, and weighted ratio methods. These traditional approaches often struggle with capturing complex interaction effects and face optimization limitations.

Compared to orthogonal design, which relies on predefined levels and fixed experimental conditions, the proposed method significantly enhances the experimental efficiency and prediction accuracy. Orthogonal design methods require multiple trials to optimize factor levels and are limited in their ability to account for complex, nonlinear interactions among drugs. In contrast, the neural network combined with the compartment model dynamically learns and predicts these interactions, effectively reducing experimental repetitions while improving precision. For example, while orthogonal design identified the optimal ratios through systematic trials, the proposed method leveraged existing data to predict the optimal ratios directly, demonstrating higher adaptability and robustness under varying experimental conditions. Specifically, orthogonal design required at least nine experimental groups (e.g., L9 matrix) to identify the most effective combination ratios, while the proposed method achieved comparable results using only three experimental groups by utilizing historical data to train the model. Furthermore, the proposed method accurately captured nonlinear interactions, such as the synergistic effect of combining *M*_1_, *M*_2_, and *PHB*, which orthogonal design typically failed to address due to its linear framework. This ability to dynamically incorporate new data further underscores its superiority in scenarios with evolving experimental conditions or limited resources.

However, to achieve the clinical translation of these findings, it is necessary to explore the challenges and pathways from animal experiments to human clinical trials. While this study demonstrated efficacy in animal models, translating these results to human trials requires addressing additional complexities. For instance, interindividual variability in metabolism, immune responses, and drug interactions necessitate further validation through pharmacokinetic and pharmacodynamic studies across diverse populations. Moreover, regulatory challenges, such as determining safety thresholds and minimizing potential side effects, must be navigated to ensure clinical applicability.

Future research will focus on expanding this approach to study more complex drug combinations and validate the optimized component ratios in combined treatments with different drugs. Incorporating physiologically based pharmacokinetic (PBPK) modeling could refine predictions by accounting for individual-specific parameters, such as age, gender, and comorbidities. This approach would not only enhance the applicability of the findings to clinical scenarios but also accelerate the translation of preclinical results into personalized combination therapies, ultimately advancing patient treatment outcomes by improving efficacy and reducing toxicity.

## Figures and Tables

**Table 1:** Plasma concentration of *PHB*, MES reaction of mice, and inhibition rate under extravascular *PHB* administration alone.

Time/h	*ρ_PHB_*/(mg·L^−1^)	MES (*y*/*n*) ^a^	Inhibition Rate/%
0.17	2.054 ± 0.261	1/4	25
0.5	2.066 ± 0.797	2/6	33
1	3.321 ± 1.422	4/5	80
1.5	2.959 ± 0.725	4/6	67
4	2.973 ± 1.112	4/6	67
7	1.751 ± 0.311	4/6	67
12	0.862 ± 0.457	2/4	50
24	0.11 ± 0.07	1/5	20

*ρ*: plasma concentration. ^a^—in the MES test, *y* represents the number of mice showing convulsive reactions (indicating generalized convulsions), which suggests that the drug is ineffective; *n* represents the total number of experimental mice, indicating that the drug is effective. The same applies to the following cases.

**Table 2:** Plasma concentration of each component, MES reaction of mice, and inhibition rate under the combined administration of extravascular *PHB*, *M*_1_, and *M*_2_.

**Time/h**	***ρM*_1_/** **(mg·L^−1^)**	***ρM*_2_/** **(mg·L^−1^)**	***ρ_PHB_*/** **(mg·L^−1^)**	**MES (*y/n*)**	**Inhibition Rate/%**
0.17	5.37 ± 5.07	0.48 ± 0.66	3.38 ± 1.80	1/6	17
0.5	5.92 ± 3.43	0.28 ± 0.22	3.32 ± 1.18	2/6	33
1	5.11 ± 1.90	0.44 ± 0.34	3.40 ± 6.56	4/5	80
1.5	4.02 ± 1.88	0.33 ± 0.39	3.49 ± 2.00	5/8	63
4	3.65 ± 1.35	0.28 ± 0.35	2.96 ± 1.05	5/7	71
7	3.39 ± 3.06	0.03 ± 0.05	1.75 ± 1.02	5/7	71
12	3.17 ± 1.82	0.03 ± 0.01	1.70 ± 0.57	4/5	80
24	0.34 ± 0.14	0.00 ± 0.00	0.30 ± 0.27	3/6	50

**Table 3:** Plasma concentration of each component, MES reaction, and inhibition rate after data expansion.

Time/h	*ρM*_1_/ (mg·L^−1^)	*ρM*_2_/ (mg·L^−1^)	*ρ_PHB_*/ (mg·L^−1^)	MES (*n*/*y*)	Inhibition Rate/%
0.17	5.10 ± 3.50	0.45 ± 0.48	3.13 ± 1.30	11/94	11.7
0.5	5.50 ± 2.50	0.50 ± 0.35	3.33 ± 1.01	36/96	37
1	4.70 ± 0.85	0.35 ± 0.33	2.33 ± 1.91	67/80	84
1.5	4.00 ± 1.70	0.37 ± 0.35	2.56 ± 1.33	83/133	62.5
4	3.60 ± 1.15	0.40 ± 0.38	3.11 ± 0.89	87/122	71
7	3.40 ± 2.40	0.37 ± 0.32	1.83 ± 0.76	77/101	77
12	0.30 ± 0.25	0.32 ± 0.33	2.33 ± 0.40	59/79	75
24	0.32 ± 0.09	0.02 ± 0.01	0.34 ± 0.18	46/85	54

**Table 4:** Inhibition rates and *CI* corresponding to the plasma concentrations of each component after data.

Time/h	*ρM*_1_/ (mg·L^−1^)	*ρM*_2_/ (mg·L^−1^)	*ρ_PHB_*/ (mg·L^−1^)	Inhibition Rate	Combination Index
0.17	5.10 ± 3.50	0.45 ± 0.48	3.13 ± 1.30	11.7%	48.0574
0.5	5.50 ± 2.50	0.50 ± 0.35	3.33 ± 1.01	37%	10.8582
1	4.70 ± 0.85	0.35 ± 0.33	2.33 ± 1.91	84%	0.91279
1.5	4.00 ± 1.70	0.37 ± 0.35	2.56 ± 1.33	62.5%	0.98306
4	3.60 ± 1.15	0.40 ± 0.38	3.11 ± 0.89	71%	0.46805
7	3.40 ± 2.40	0.37 ± 0.32	1.83 ± 0.76	77%	0.19855
12	0.30 ± 0.25	0.32 ± 0.33	2.33 ± 0.40	75%	0.07089
24	0.32 ± 0.09	0.02 ± 0.01	0.34 ± 0.18	54%	0.023674

**Table 5:** Plasma concentration of each component, MES reaction, and inhibition rate after the efficacy was improved.

Time/h	*ρM*_1_/ (mg·L^−1^)	*ρM*_2_/ (mg·L^−1^)	*ρ_PHB_*/ (mg·L^−1^)	MES (*n*/*y*)	Inhibition Rate /%
0.17	2.24 ± 1.55	0.22 ± 0.23	1.68 ± 0.67	15/96	16
0.5	3.70 ± 2.03	0.18 ± 0.10	1.51 ± 0.36	39/96	41
1	3.67 ± 1.13	0.23 ± 0.08	1.79 ± 2.81	71/80	89
1.5	1.38 ± 0.64	0.23 ± 0.21	0.97 ± 0.47	90/133	68
4	1.11 ± 0.34	0.19 ± 0.18	1.13 ± 0.32	93/121	76
7	0.98 ± 0.67	0.22 ± 0.04	0.80 ± 0.32	83/101	82
12	0.86 ± 0.52	0.02 ± 0.04	1.10 ± 0.25	63/96	80
24	0.35 ± 0.52	0.01 ± 0.04	0.34 ± 0.39	50/85	59

**Table 6:** Evaluation indexes of different dose–effect models after the efficacy was improved.

Model	Precision	Recall	F1-Score	Accuracy
HO-1DCNN	0.91	0.93	0.92	0.96
1DCNN	0.89	0.90	0.88	0.92
CatBoost	0.87	0.88	0.86	0.90

**Table 7:** Performance indexes of different models.

Model	Prediction Accuracy	Prediction Time/min
HO-1DCNN	0.91	0.50
WOA-1DCNN	0.87	0.62
SSA-1DCNN	0.89	0.61
GWO-1DCNN	0.83	0.66
Adam-1DCNN	0.85	0.70
RMSprop-1DCNN	0.84	0.84

**Table 8:** Plasma concentration, inhibition rate, and *CI* after the efficacy enhancement.

Time/h	*ρM*_1_/ (mg·L^−1^)	*ρM*_2_/ (mg·L^−1^)	*ρ_PHB_*/ (mg·L^−1^)	Inhibition Rate	Combination Index
0.17	2.24 ± 1.55	0.22 ± 0.23	1.68 ± 0.67	16.7%	12.0210
0.5	3.70 ± 2.03	0.18 ± 0.10	1.51 ± 0.36	41%	1.74799
1	3.67 ± 1.13	0.23 ± 0.08	1.79 ± 2.81	89%	0.03249
1.5	1.38 ± 0.64	0.23 ± 0.21	0.97 ± 0.47	67.5%	0.20925
4	1.11 ± 0.34	0.19 ± 0.18	1.13 ± 0.32	76%	0.10462
7	0.98 ± 0.67	0.22 ± 0.04	0.80 ± 0.32	82%	0.02495
12	0.86 ± 0.52	0.02 ± 0.04	1.10 ± 0.25	80%	0.04890
24	0.35 ± 0.52	0.01 ± 0.04	0.34 ± 0.39	59%	0.02729

**Table 9:** Plasma concentration of each component, clearance count ratio, and clearance rate under the combined administration of *Bre*, *Scu*, and *His* at equal plasma concentrations.

Time/h	*ρBre*/ (ug·mL^−1^)	*ρScu*/ (ug·mL^−1^)	*ρHis*/ (ug·mL^−1^)	MES (*y/n*)	Inhibition Rate/%
0.25	28.11 ± 8.38	53.00 ± 18.60	20.74 ± 10.90	1/6	17
0.5	71.09 ± 24.29	80.40 ± 18.42	22.82 ± 10.03	2/6	33
1	50.18 ± 15.79	50.09 ± 21.87	38.77 ± 13.35	7/11	63
2	94.34 ± 27.57	149.30 ± 59.10	37.93 ± 17.63	4/6	67
4	33.98 ± 15.44	263.45 ± 56.68	52.01 ± 18.72	5/6	83
8	25.83 ± 12.53	84.31 ± 54.39	29.16 ± 13.90	3/10	30
24	10.00 ± 3.98	11.63 ± 4.09	23.90 ± 15.25	1/5	20

**Table 10:** Plasma Concentration, MES reaction, and inhibition rate of *Erigeron breviscapus* after data expansion.

Time/h	*ρBre*/ (ug·mL^−1^)	*ρScu*/ (ug·mL^−1^)	*ρHis*/ (ug·mL^−1^)	MES (*y/n*)	Inhibition Rate/%
0.25	29.38 ± 6.49	52.22 ± 14.22	21.76 ± 8.49	20/120	16.7
0.5	65.56 ± 22.42	79.62 ± 16.59	24.38 ± 7.69	60/180	33.3
1	53.09 ± 15.36	59.09 ± 20.92	35.96 ± 10.97	69/109	63.3
2	89.20 ± 21.17	141.02 ± 43.56	36.72 ± 13.23	119/177	67.2
4	32.30 ± 11.92	266.54 ± 46.20	50.89 ± 12.78	150/180	83.3
8	27.37 ± 10.62	83.55 ± 44.07	31.26 ± 14.21	54/174	31
24	10.31 ± 3.33	11.37 ± 3.41	21.69 ± 12.67	33/153	21.5

**Table 11:** Inhibition rates and *CI* corresponding to the plasma concentrations of each component of *Erigeron breviscapus* after data expansion.

Time/h	*ρBre/* (mg·L^−1^)	*ρScu/* (mg·L^−1^)	*ρHis/* (mg·L^−1^)	Inhibition Rate	Combination Index
0.25	29.38 ± 6.49	52.22 ± 14.22	21.76 ± 8.49	16.7	1.99573
0.5	65.56 ± 22.42	79.62 ± 16.59	24.38 ± 7.69	33.3	0.85689
1	53.09 ± 15.36	59.09 ± 20.92	35.96 ± 10.97	63.3	0.24518
2	89.20 ± 21.17	141.02 ± 43.56	36.72 ± 13.23	67.2	0.25751
4	32.30 ± 11.92	266.54 ± 46.20	50.89 ± 12.78	83.3	0.16750
8	27.37 ± 10.62	83.55 ± 44.07	31.26 ± 14.21	31	0.892286
24	10.31 ± 3.33	11.37 ± 3.41	21.69 ± 12.67	21.5	0.58988

**Table 12:** Plasma concentration of each component, MES reaction, and inhibition rate of *Erigeron breviscapus* after the efficacy was improved.

Time/h	*ρBre*/ (ug·mL^−1^)	*ρScu*/ (ug·mL^−1^)	*ρHis*/ (ug·mL^−1^)	MES (*y/n*)	Inhibition Rate/%
0.25	15.47 ± 0.78	38.78 ± 3.34	5.902 ± 0.66	26/120	21.6
0.5	30.06 ± 2.67	35.29 ± 2.78	7.06 ± 0.78	70/180	38.7
1	21.65 ± 2.09	22.14 ± 2.66	21.08 ± 1.96	74/109	68.1
2	34.54 ± 3.77	49.70 ± 5.79	17.55 ± 1.88	129/177	72.7
4	18.81 ± 1.87	68.05 ± 3.47	26.80 ± 3.34	158/180	87.8
8	13.77 ± 1.30	53.30 ± 6.11	10.15 ± 1.30	63/174	36.1
24	3.92 ± 0.39	7.54 ± 0.86	15.76 ± 0.81	40/153	26.3

**Table 13:** Plasma concentration, inhibition rate, and *CI* of *Erigeron breviscapus* after the efficacy enhancement.

Time/h	*ρBre*/ (ug·mL^−1^)	*ρScu*/ (ug·mL^−1^)	*ρHis*/ (ug·mL^−1^)	Inhibition Rate	Combination Index
0.25	15.47 ± 0.78	38.78 ± 3.34	5.902 ± 0.66	21.6%	0.69369
0.5	30.06 ± 2.67	35.29 ± 2.78	7.06 ± 0.78	38.7%	0.24437
1	21.65 ± 2.09	22.14 ± 2.66	21.08 ± 1.96	68.1%	0.11369
2	34.54 ± 3.77	49.70 ± 5.79	17.55 ± 1.88	72.7%	0.09132
4	18.81 ± 1.87	68.05 ± 3.47	26.80 ± 3.34	87.8%	0.06527
8	13.77 ± 1.30	53.30 ± 6.11	10.15 ± 1.30	36.1%	0.31708
24	3.92 ± 0.39	7.54 ± 0.86	15.76 ± 0.81	26.3%	0.29406

## Data Availability

The original contributions presented in this study are included in the article and the data that support the findings of this study are available from the corresponding author, [Y.L.], upon reasonable request.
